# Emergence and Transmission Pathways of Rapidly Evolving Evolutionary Branch C4a Strains of Human Enterovirus 71 in the Central Plain of China

**DOI:** 10.1371/journal.pone.0027895

**Published:** 2011-11-18

**Authors:** Yong Zhang, Jitao Wang, Wanshen Guo, Haiyan Wang, Shuangli Zhu, Dongyan Wang, Ruyin Bai, Xingle Li, Dongmei Yan, Huiling Wang, Yan Zhang, Zhen Zhu, Xiaojuan Tan, Hongqiu An, Aiqiang Xu, Wenbo Xu

**Affiliations:** 1 WHO WPRO Regional Polio Reference Laboratory and State Key Laboratory for Molecular Virology and Genetic Engineering, National Institute for Viral Disease Control and Prevention, Chinese Center for Disease Control and Prevention, Beijing, People's Republic of China; 2 Taiyuan Center for Disease Control and Prevention, Taiyuan City, Shanxi Province, People's Republic of China; 3 Henan Center for Disease Control and Prevention, Zhengzhou City, Henan Province, People's Republic of China; 4 Shandong Provincial Key Laboratory of Infectious Disease Control and Prevention, Shandong Center for Disease Control and Prevention, Jinan City, Shandong Province, People's Republic of China; 5 Taishan Medical University, Taishan City, Shandong Province, People's Republic of China; University of Hong Kong, Hong Kong

## Abstract

**Background:**

Large-scale outbreaks of hand, foot, and mouth disease (HFMD) occurred repeatedly in the Central Plain of China (Shandong, Anhui, and Henan provinces) from 2007 until now. These epidemics have increased in size and severity each year and are a major public health concern in mainland China.

**Principal Findings:**

Phylogenetic analysis was performed and a Bayesian Markov chain Monte Carlo tree was constructed based on the complete *VP1* sequences of HEV71 isolates. These analyses showed that the HFMD epidemic in the Central Plain of China was caused by at least 5 chains of HEV71 transmission and that the virus continued to circulate and evolve over the winter seasons between outbreaks. Between 1998 and 2010, there were 2 stages of HEV71 circulation in mainland China, with a shift from evolutionary branch C4b to C4a in 2003–2004. The evolution rate of C4a HEV71 was 4.99×10^-3^ substitutions per site per year, faster than the mean of all HEV71 genotypes. The most recent common ancestor estimates for the Chinese clusters dated to October 1994 and November 1993 for the C4a and C4b evolutionary branches, respectively. Compared with all C4a HEV71 strains, a nucleotide substitution in all C4b HEV71 genome (A to C reversion at nt2503 in the *VP1* coding region, which caused amino acid substitution of VP1–10: Gln to His) had reverted.

**Conclusions:**

The data suggest that C4a HEV71 strains introduced into the Central Plain of China are responsible for the recent outbreaks. The relationships among HEV71 isolates determined from the combined sequence and epidemiological data reveal the underlying seasonal dynamics of HEV71 circulation. At least 5 HEV71 lineages circulated in the Central Plain of China from 2007 to 2009, and the Shandong and Anhui lineages were found to have passed through a genetic bottleneck during the low-transmission winter season.

## Introduction

Human enteroviruses (HEVs) belong to the family *Picornaviridae* and are divided into 4 species: HEV-A, HEV-B, HEV-C, and HEV-D [1]. The HEV-A species now includes 18 serotypes of coxsackievirus A (CVA; serotypes 2–8, 10, 12, 14, and 16) and HEV serotypes 71, 76, 89–92 [2], and 114 [3]. Hand, foot, and mouth disease (HFMD) is an acute enterovirus infection most often caused by HEV-A species. Human enterovirus 71 (HEV71) and coxsackievirus A16 (CVA16) are the 2 major causative agents of HFMD, and the co-circulation of both pathogens has frequently been described [4], [5].

HFMD is a common infectious illness in young children, particularly those less than 5 years of age. The illness typically occurs as outbreaks and is characterized by mucocutaneous papulovesicular lesions on the hands, feet, mouth, and buttocks. HFMD usually resolves spontaneously. CVA16-associated HFMD is milder than that caused by HEV71 and has a much lower incidence of severe complications, including death [6]. However, a variety of neurological diseases, including aseptic meningitis, encephalitis, and poliomyelitis-like paralysis, can sometimes arise, particularly when HEV71 is the causative agent [7], [8], [9], [10].

In recent years, numerous large outbreaks of HEV71-associated HFMD with high morbidity and mortality have occurred in eastern and southeastern Asian countries and regions, including Singapore [11], South Korea [12], Malaysia [13], Japan [14], Vietnam [15], Mainland China [16], [17], and Taiwan [18], [19]. This phenomenon has increased the research interest in HEV71, leading to extensive nucleotide sequencing and genotype description [20], [21].

The earliest known case of HFMD in China was diagnosed in Shanghai in 1981 and was followed by the reports of HFMD in most of the Chinese provinces, but at that time the disease appeared only sporadically and there were no large-scale outbreaks or patient deaths reported. In 2007, HEV71 began to cause large-scale epidemics of HFMD associated with acute neurological disease in the Central Plain of China [16], and since then the outbreak pattern has repeated each year, with the reported number of HFMD patients with severe or fatal disease increasing year by year [17], [22]. These increasingly large and severe HFMD epidemics are a major public health concern in mainland China, and the continuing lack of effective prevention and treatment measures such as vaccines or antiviral drugs makes HFMD outbreaks public health emergencies.

It has been confirmed that subgenotype C4 has been the sole viral genetic lineage circulating in mainland China since 1998 [16]. The large HFMD outbreaks with fatal neurological complications that have occurred since 2007 are mainly due to subgenotype C4a of HEV71, and as these outbreaks might be a threat to public health in China, it would be worthwhile to perform molecular characterization of HEV71 and determine its evolutionary pathway in order to understand the annual outbreaks in the Central Plain of China.

Since the epidemic developed over a relatively short time span and is associated with high morbidity and mortality, HEV71-associated HFMD has received considerable attention from clinicians and public health officials, and HFMD was classified as a category C notifiable infectious disease by the Ministry of Health of P. R. China on May 2, 2008. As HFMD is a category C notifiable infectious disease, suspected cases are to be reported to the National Notifiable Diseases Reporting System (NNDRS) within 24 h of detection. The information collected includes the name, age, sex, residence, date of onset, vaccination status, and occupation of each patient. Cases are not confirmed by laboratory testing. NNDRS data are submitted monthly to the Chinese Center for Disease Control and Prevention as standardized aggregate data tables. The numbers and descriptions of HFMD cases and deaths in this report were based on data submitted to the NNDRS.

The intensive surveillance for HEV71 circulation maintained by mainland China during and after the 2007 outbreak permitted a detailed analysis of the outbreak by molecular epidemiological methods. To address the nature and evolutionary pathway of outbreak-associated HEV71, we compared the nucleotide sequences of the outbreak isolates with each other and with the sequences of other contemporary HEV71 isolates. Because significant evolution of the viral RNA genome had occurred during the 4-year outbreak, individual chains of virus transmission could be visualized as separate genetic lineages. We were able to use the combined epidemiological and sequencing data to obtain a high-resolution view of the pathways of HEV71 transmission during the outbreaks.

## Results

### Five lineages of HEV71 circulated in the Central Plain of China during the outbreaks

**Figure 1:**
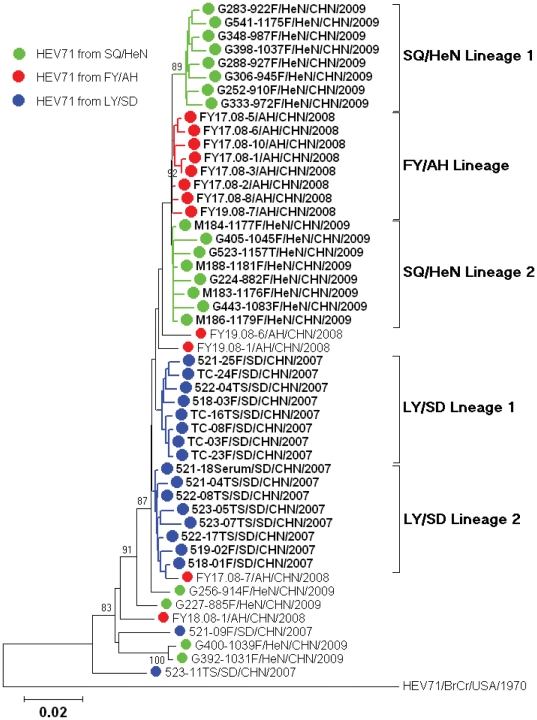
Five lineages of HEV71 circulated in the Central Plain of China. Abbreviations of Chinese cities/provinces: LY/SD, Linyi/Shandong; FY/AH, Fuyang/Anhui; SQ/HeN, Shangqiu/Henan.

The complete *VP1* sequences of 50 HEV71 isolates from HFMD patients in Shandong (18 from the GenBank database), Anhui (12 from the GenBank database), and Henan (20 from this study) provinces of China were used for phylogenetic analysis (Table S1, Figure 1). As shown in the phylogenetic tree, the isolates could be divided into at least 5 lineages with distinct local characteristics. The Shandong strains (isolated from the 2007 Linyi outbreak) [16] belonged to LY/SD lineages 1 and 2, the Anhui strains (isolated from the 2008 Fuyang outbreak) [17] to the third lineage, FY/AH, and the Henan strains (isolated from the 2009 Shangqiu outbreak) to the fourth and fifth lineages, SQ/HeN lineages 1 and 2. A 0.9–2.3% nucleotide divergence was found among these 5 lineages, suggesting that the HFMD epidemic in the Central Plain of China was caused by at least 5 chains of virus transmission chains of HEV71 that continued to evolve and circulate during the low-transmission winter seasons. It was noteworthy that some single sporadic isolates from the 3 provinces could not be assigned to any lineage (Figure 1), suggesting that they were imported rather than derived from the main chains of virus transmission.

### Two stages of HEV71 circulation have occurred in mainland China since 1998

**Figure 2:**
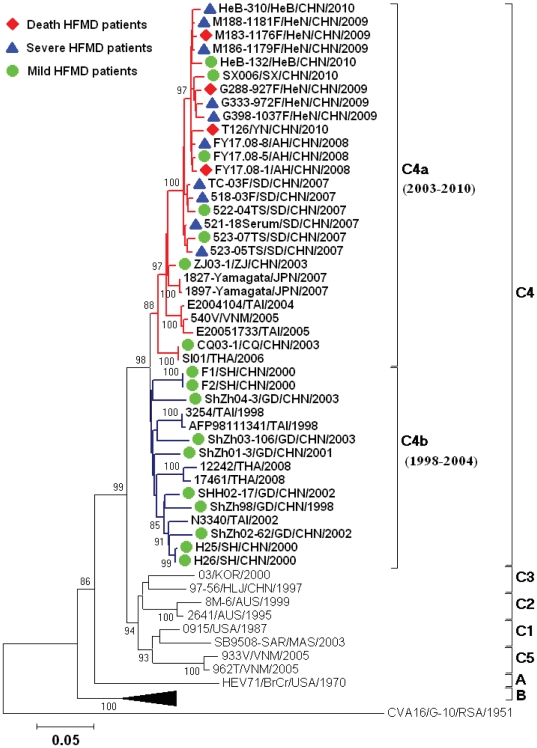
Sustained transmission of the C4a evolutionary branch in the Central Plain of China. Abbreviations of Chinese provinces: SD, Shandong; AH, Anhui; HeN, Henan; YN, Yunnan; SX, Shanxi; HeB, Hebei; ZJ, Zhejiang; CQ, Chongqing, SH, Shanghai; GD, Guangdong.

The complete *VP1* sequences were used to describe the phylogenetic relationships between the HEV71 strains by defining different genotypes of these viruses. To determine the molecular epidemiology of HEV71 strains associated with the HFMD epidemic in the Central Plain of China, a phylogenetic dendrogram was constructed from 15 selected HEV71 isolates from the Central Plain of China (3 strains randomly selected from each lineage based on their genetic relationships), 16 HEV71 isolates from other parts of mainland China, and 19 HEV71 strains from outside of mainland China that represented known genotypes (A to C) [20] (Table S1, Figure 2).

As in a previous study [16], [20], all HEV71 isolates could be grouped into genotypes A, B, and C. Furthermore, the HEV71 isolates from the Central Plain of China clearly belonged to subgenotype C4, as did those collected from mainland China from 1998 to 2010. The sequences in subgenotype C4 could be further divided into C4a and C4b evolutionary branches, and the mean *p*-distance between these 2 evolutionary branches was 7.1% (Figure 2). The C4b evolutionary branch was found in Shenzhen and Shanghai during 1998–2004 as well as in Taiwan and Thailand. The mean *p*-distance within evolutionary branch C4b was 5.1%. Evolutionary branch C4a was found in mainland China beginning in 2003 and in Taiwan, Japan, Vietnam, and Thailand during the same period. The mean *p*-distance within evolutionary branch C4a was 2.9%.

The molecular epidemiology of HEV71 in mainland China during the last 13 years reflects the pattern of endemic circulation of subgenotype C4 viruses. There were 2 stages of HEV71 circulation in mainland China between 1998 and 2010, and a shift from evolutionary branch C4b to C4a occurred during 2003–2004. In the first stage, from 1998 to 2004, HEV71 isolates from Shenzhen and Shanghai belonged to evolutionary branch C4b, which was seldom associated with high morbidity and mortality and has now disappeared. Almost concurrent with the disappearance of evolutionary branch C4b, evolutionary branch C4a emerged and has spread since 2003. It continued to evolve for 4 years before beginning to cause high morbidity and mortality in 2007 and has become the predominant HEV71 type circulating in mainland China (Figure 2).

### Genetic characterizations and recombinant features of Chinese C4a and C4b HEV71 strains

The full-length genome sequences of 12 China C4a HEV71 strains were determined, and were similar to other reported genomes of HEV71, with 7,404–7,406 nucleotides, including 5′ untranslated region (UTR) of 741–743 nucleotides, a single open reading frame (ORF) of 6,579 nucleotides encoding a single polyprotein of 2,193 amino acid, and a 3′ UTR of 82 nucleotides preceding the poly (A) tract. All these sequences shared 97.31–99.57% nucleotide sequence identities with each other, validating the circulation of C4a HEV71 in the central plain of China. The sequence homologies between 12 China C4a HEV71 strains and the C4b HEV71 strain (SHZH98 strain) were 91.1–91.9% in the full-length genome sequence and 91.1–91.7%, 89.8–91.1%, and 90.9–91.9% in the *P1*, *P2*, and *P3* coding region sequences, respectively. These results revealed that China C4a and C4b HEV71 strains have the similar genetic structures.

**Figure 3:**
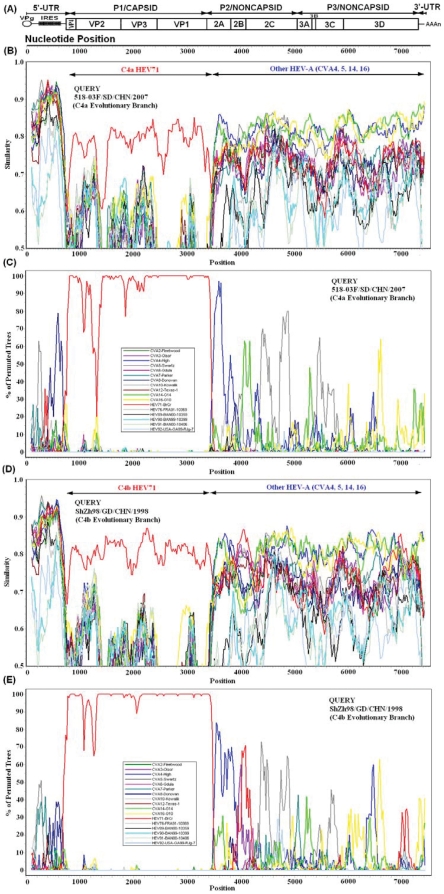
Similarity plot and bootscan analysis of whole genomes of C4b HEV71 strains. Similarity plot and bootscan analysis of complete genomes of C4a and C4b HEV71 strains using a sliding window of 200 nt moving in 20-nt steps. For each plot, the names of viruses of the query sequence were indicated in the upper left corner, and for each bootscan analysis, the names of viruses of the query sequence were indicated in the upper right corner.

Nucleotide acid sequences and deduced amino acid sequences within the *P1* capsid coding region were compared, and genomic sequences of C4b HEV71 showed that their genome were co-linear with that of the C4a HEV71, nucleotide substitutions were scattered throughout the genome. It is noteworthy that, compared with all C4a HEV71, a nucleotide substitution in all C4b HEV71 genome (A to C reversion at nt2503 in the *VP1* coding region, which caused amino acid substitution of VP1–10: Gln to His) had reverted. Similarity plot and bootscan analyses revealed recombination between subgenotype C4a or C4b HEV71 and HEV-A at the *2A*–*2B* junction. The C4a and C4b HEV71 were all identified as a HEV71 capsid sequence that containing an unidentified sequence in the *P2* and *P3* coding region that was apparently not related to those of HEV71 strains (Figure 3). Comparison of the *P2* and *P3* coding region sequences of the C4a and C4b HEV71 strains with those of the certain prototype strains of HEV-A, B, C, and D revealed no sequence match above 88.3%, and showed higher similarity to HEV-A than to HEV-B, C, and D. In addition, the deduced amino acid sequence of the recombinant noncapsid sequences of the C4a and C4b HEV71 strains showed a high identity with HEV-A, especially those of prototype CVA16 (97.7%), prototype CVA14 (97.4%), and prototype CVA4 (97.1%). These results suggest that the recombinant noncapsid sequences might be classified into a HEV-A phylogeny (Figure 3), and indicate that the recombination events occurred before the differentiation of C4a and C4b evolutionary branch HEV71 from a common ancestor.

### Pathways of HEV71 evolution and transmission during the repeated outbreaks

**Figure 4:**
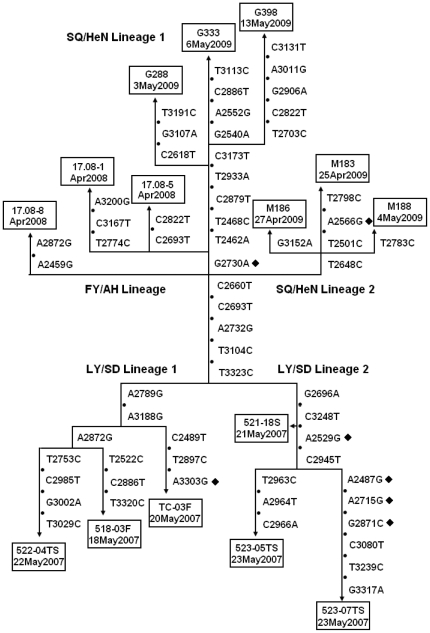
Tree summarizing evolutionary relationships among outbreak isolates based upon differences in *VP1* nucleotide sequences. The branch structure of the tree was constructed under the assumption that each substitution occurred only once, and the tree was rooted to the 2007 Shandong outbreak isolates. Vertical branches are scaled to the number of nucleotide differences between *VP1* sequences. The *VP1* nucleotide substitutions that encode amino acid changes are indicated by symbol“⧫” The dates shown are those on which the clinical samples were taken. Abbreviations of Chinese cities/provinces: LY/SD, Linyi/Shandong; FY/AH, Fuyang/Anhui; SQ/HeN, Shangqiu/Henan.

The pattern of *VP1* sequence evolution in the genetic data from the outbreak isolates enabled us to reconstruct the main pathways of HEV71 transmission. The observed *VP1* sequence differences among the recent outbreak isolates are represented in a tree (Figure 4) constructed under the assumption that each nucleotide substitution had occurred only once. The dates shown on the tree are those on which the samples were collected. Branch nodes represent the inferred *VP1* sequences of intermediate viruses that were usually not observed as isolates. The vertical length of each segment is proportional to the number of nucleotide substitutions that define that segment.

**Figure 5:**
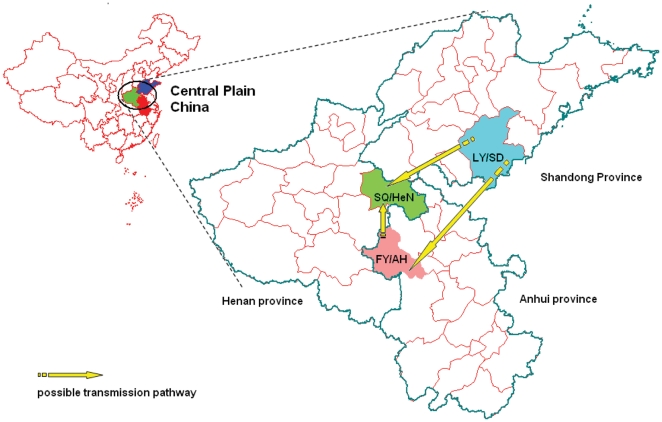
The locations and possible HEV71 transmission pathways of the HFMD outbreaks in the Central Plain of China during 2007–2009. HFMD outbreaks with high morbidity and mortality occurred in 2007, 2008, and 2009 and began in Linyi City in Shandong province (blue), Fuyang City in Anhui province (green), and Shangqiu City in Henan province (red), respectively. The possible transmission pathways of HEV71 are indicated by yellow arrows. Abbreviations of Chinese cities/provinces: LY/SD, Linyi/Shandong; FY/AH, Fuyang/Anhui; SQ/HeN, Shangqiu/Henan.

The pathways of *VP1* evolution shown in the tree also trace the major chains of HEV71 transmission during the outbreaks (Figure 5). The tree is shown as having 2 alternative roots representing the sequences of the 2007 Shandong isolates (LY/SD Lineages 1 and 2) (Figure 4). The sequences of all 2008 Anhui and 2009 Henan outbreak viruses differed from those of the hypothetical founder (2007 Shandong isolates) by the substitutions T3323C, T3104C, A2732G, C2693T, and C2660T (Figure 4).

After 2008, the common pathway diverged into multiple independent lineages (Figure 4). In the FY/AH lineage, the first branch (identified by substitutions A2459G and A2872G) observed to have split from the main transmission pathway terminates with the isolate 17.08-8/FY/AN/CHN, with 2 more branches from the main pathway at the next node leading to isolates 17.08-1/FY/AN/CHN and 17.08-5/FY/AN/CHN. In SQ/HeN lineage 2, there is a main branch that splits into 3 sub-branches terminating with isolates M183/SQ/HeN/CHN, M186/SQ/HeN/CHN, and M188/SQ/HeN/CHN (Figure 4).

Above the node at which the FY/AH lineage diverged, the main transmission pathway is identified by the substitutions T2462A, T2468C, C2879T, and T2933A. Isolates G288/SQ/HeN/CHN, G333/SQ/HeN/CHN, and G398/SQ/HeN/CHN are derived from the main transmission pathway and form a close genetic cluster (SQ/HeN lineage 1) near the top of the tree.

**Figure 6:**
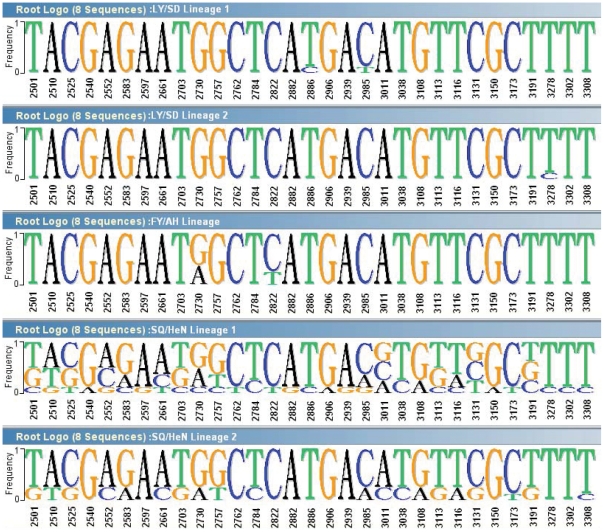
Sequence logo showing nucleotide acid diversity in the *VP1* region sequences among these 5 lineages of HEV71. Only mutated positions were shown. More mutated positions occurred in the SQ/HeN lineages than FY Lineage and LY/SD Lineages. Sequence logo was constructed using Phylo-mLogo [44].

This was also seen in our new pairwise contrast logo visualizations in Figure 6. In the 5 lineages dataset (each 8 sequences), the frequency of each base at each position was determined, and the nucleotide acid changes were identified in 31 positions in *VP1* region (Figure 6). There is a growing trend in nucleotide acid changes, that is, in LY/SD lineages 1 and 2, only a small numbers of nucleotide acid changes were found, more changes occurred in FY/AH lineage, and the largest nucleotide acid changes were found in SQ/HeN lineage 1 and 2.

### Origin, evolutionary rate, and molecular clock phylogeny

The sequences of the complete *VP1* region of the representative subgenotype C4 HEV71 isolates from mainland China (n = 39, including 22 sequences from the Central Plain of China), subgenotype C4 HEV71 isolates from elsewhere (n = 11), and other genotype HEV71 isolates (n = 19, from 9 countries) (Table S1) were analyzed for divergence time and substitution rate estimation using the Bayesian Markov chain Monte Carlo (MCMC) method. Different models were used for data analysis, and the uncorrelated log-normal distributed (UCLD) with exponential growth (EG) model was found to fit our data best, while the HKY and GTR nucleotide substitution models had no significant impact on the analysis (Table 1). The coefficients of variation of the evolutionary rates among the branches were 0.44 (95% highest posterior density [HPD] 0.27–0.63) and 0.44 (95% HPD, 0.26– 0.63) when estimated by the Hasegawa-Kishino-Yano (HKY) model and the general time reversible (GTR) model, respectively, indicating that rate heterogeneity exists among the different branches.

**Table 1:** Origins and evolutionary rates inferred from the Bayesian MCMC method applied to the complete *VP1* region of HEV71.

Parameter	Mean value of the parameter (95% HPD) as determined by:	
	HKY+UCLD+EG	GTR+UCLD+EG
*t*_MRCA_ HEV71	1911.11 (1875.11–1942.11)	1909.8 (1874.2–1942.2)
*t*_MRCA_ C4a evolutionary branch	1994.10 (1992.4–1997.2)	1994.10 (1992.3–1996.12)
*t*_MRCA_ C4b evolutionary branch	1994.3 (1991.9–1996.6)	1993.11 (1991.5–1996.6)
Mean evolutionary rate (10^−3^ substitutions/site·y) a	3.28×10^−3^ (2.52×10^−3^–4.15×10^−3^)	3.18×10^−3^ (2.44×10^−3^–3.98×10^−3^)
C4a evolutionary rate (10^−3^ substitutions/site·y) a	4.99×10^−3^ (3.57×10^−3^–6.57×10^−3^)	4.97×10^−3^ (3.55×10^−3^–6.44×10^−3^)
Coefficient of variation	0.44 (0.27–0.63)	0.44 (0.26–0.63)

a The rate of molecular evolution is given as numbers of nucleotide substitutions per site per year.

**Figure 7:**
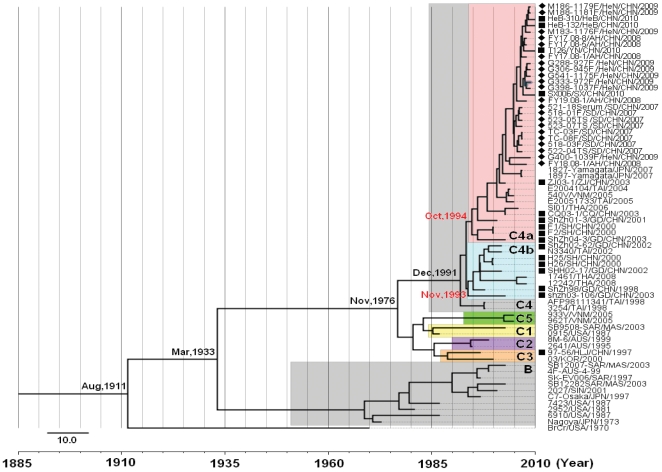
MCMC tree of the complete *VP1* region sequence of HEV71 strains found worldwide visualized using FigTree. The width of a branch reflects the evolutionary rate of the individual sequences and their reconstructed ancestors. HEV71 isolates collected in mainland China from 1998 to 2010 segregated into 2 clusters (C4a and C4b evolutionary branches). The most recent common ancestor (*t*_MRCA_) estimates for the Chinese clusters were dated to Oct, 1994 (95% HPD, Mar, 1992–Dec, 1996) and Nov, 1993 (95% HPD, May, 1991–Jun, 1996) for the C4a and C4b evolutionary branches, respectively. Diamonds indicate HEV71 isolates from the Central Plain of China, and solid squares indicate HEV71 isolates from other parts of mainland China.

In the molecular clock Bayesian MCMC tree (Figure 7), total 38 C4a and 9 C4b HEV71 strains were analyzed, and the HEV71 isolates from mainland China belonged to either the C4a or the C4b evolutionary branch. All recent HEV71 isolates belonged to the C4a evolutionary branch, which was present in all provinces of mainland China. Viruses from the C4b evolutionary branch (such as isolates from the cities of Shenzhen and Shanghai) were isolated during 1998–2003, and 8 years have passed since the last isolation of this branch. In contrast, recent isolates from these cities are located in the C4a evolutionary branch, indicating that the older strains may have been eliminated in mainland China. However, it is interesting that C4b HEV71 which has high nucleotide homology with strains of Shenzhen strains was found in Thailand in 2008, which indicated that C4b HEV71 extinct in mainland China, it spread to other countries such as Thailand and survives there.

Different evolutionary rates were observed for different branches of the tree, and the mean rate was 3.28×10^−3^ substitutions per site per year (HKY) or 3.18×10^−3^ substitutions per site per year (GTR). C4a HEV71 isolates had a mutation rate of 4.99×10^−3^ substitutions per site per year (HKY) or 4.97×10^−3^ substitutions per site per year (GTR), faster than the mean rate for all HEV71 genotypes (Table 1). The most recent common ancestor (*t*_MRCA_) of global genotype C HEV71 can be traced back to November 1976 (95% HPD, November 1967–March 1984). The *t*_MRCA_ estimates for the Chinese clusters were dated to Oct, 1994 (95% HPD, Mar, 1992–Dec, 1996) for the C4a evolutionary branch and Nov, 1993 (95% HPD, May, 1991–Jun, 1996) for the C4b evolutionary branch.

## Discussion

Before 2007, only limited HFMD outbreaks producing mild disease had been documented in China, and there was therefore no compulsory HFMD surveillance. Both the epidemiological and molecular biological data presented in our study support the recent sudden onset of outbreaks of severe HFMD due to HEV71 infection as representative of an emerging disease in China.

The protein encoded by the *VP1* coding region is considered to be the most important determinant of antigenicity [23], and analysis of the complete *VP1* sequence is considered the most rigorous method for determining the genotype of HEV and provides the most useful information for molecular epidemiology studies [20], [24], [25]. Phylogenetic classification based on the complete *VP1* region (891 bp) of HEV71 has been used to describe different genotypes of the viruses [20], and such classification has proven useful for tracking HEV71 genotypes across different temporal and geographical HFMD outbreaks [16], [21], [26].

Our study showed that different strains of HEV71 are broadly geographically distributed in mainland China and that the viruses are more genetically variable than the circulating CVA16 strains. The evolution rate of the C4a HEV71 lineages was estimated to be 4.97×10^−3^ substitutions per nucleotide per year, which was higher than the rate calculated for CVA16 (2.49×10^−3^) [27] and the average rate for all HEV71 genotypes (3.18×10^−3^) [20]. The evolution of HEV71 and that of CVA16 were similar in the sense that their prototype strains were the sole members of genotype A, and were largely replaced by modern strains due to molecular evolution. CVA16 was first identified in South Africa in 1951, and over the course of approximately 60 years of evolution all subsequent CVA16 strains except this prototype formed a single genotype (genotype B) containing 2 subgenotypes (B1 and B2) [27]. The evolution rate of HEV71 has been relatively fast in comparison: HEV71 was first identified in the USA in 1970 and diverged into 2 co-circulating genotypes (genotypes B and C) within only 40 years of evolution [20]. At present, the B genotype of HEV71 is known to contain 6 subgenotypes (B0–B5), and the C genotype of HEV71 is known to contain another 5 (C1–C5) [16], [21], [28]. Recently, based on the full-length genome sequencing, the nucleotide sequence divergence of the C4 isolates was 17–20% due to recombination when compared to other isolates of subgenotype C, which reach the cut-off divergence value of 17–22% used to designate the viral genotypes. Based on these findings, some researchers believe that HEV71 subgenotype C4 can be proposed as a novel genotype D [29], [30].

The molecular epidemiology of HEV71 from several different countries and regions showed that the patterns of HEV71 prevalence varied among different areas. In some circumstances, multiple subgenotypes of HEV71 were prevalent during the same period, while in other cases different subgenotypes of HEV71 alternated as the dominant strain in different years. For example, Taiwan experienced serial HEV71 epidemics with different genotypes predominating; subgenotype C2 was the dominant type circulating in 1998 [19], followed by subgenotype B4 in 2000–2001 [31], subgenotype C4 in 2004–2005 [32], [33], and subgenogroups of B5 and C5 in 2006–2007 [28], while the 2008 HEV71 epidemic was caused by a subgenotype B5 strain that was likely introduced to Taiwan from Southeast Asia [18]. While the situation in mainland China is very different, the molecular epidemiology of HEV71 during the last 14 years shows only subgenotype C4 viruses in endemic circulation [16]. Because the abovementioned HEV71 strains of different subgenotypes are all virulent, the genotype was not the only factor that determined HEV71 neurovirulence.

It is also interesting that the molecular epidemiological pattern of HEV71 in mainland China appears to be similar to those of the measles virus [34] and CVA16 in mainland China [27], but to be quite different from those of the rubella virus that different genotypes have co-circulated in China [35]. Virological surveillance for measles virus since 1995 shows that the genotype H1 of measles virus is the only genotypes found thus far in mainland China, genotype B1 of CVA16 has been continuously circulating in China since they were firstly detected in 1999. It is likely that viruses that have succeeded in creating a good balance between host and viruses have survived, and this phenomenon may reflect the balance between viruses and hosts.

Our molecular epidemiological studies have preliminarily described the genetic characteristics, prevalence, and dissemination of HEV71 in mainland China. All HEV71 strains that have circulated in mainland China since 1998 belonged to subgenotype C4, and although no replacement of genotypes or subgenotypes has recently occurred, a shift between 2 evolutionary branches within subgenotype C4 has been documented [16]. Evolutionary branch C4b HEV71 (which was prevalent from 1998 to 2004 but has now disappeared from mainland China), which rarely caused severe disease and death in HFMD patients, evolved into the C4a evolutionary branch (which first appeared in 2003), which showed higher morbidity and mortality and caused many severe and fetal HFMD patients. It is speculated that the neurovirulence of evolutionary branch C4a increased due to continuing evolution, as C4a evolved for 4 years after its appearance before beginning to cause large-scale outbreaks.

The reason for the epidemic of large-scale outbreaks of HFMD with increasing morbidity and mortality has been one of the most important issues in biomedical research in recent years. We speculate that both evolutionary changes in the biological characteristics of HEV71 and host factors have been very important in the emergence of epidemic HFMD [36], [37]. Evolutionary branch C4a has some key nucleotide or amino acid mutations relative to branch C4b, and these changes may be responsible for its increased neurovirulence. Coupled with circumstances such as the accumulation of a large number of HEV71-susceptible individuals, these characteristics of the emerging C4a evolutionary branch of HEV71 could have produced the unusually large range of transmission observed. However, speculation along these lines is limited by the fact that the molecular basis of the neurovirulence determinants of HEV71, unlike that of poliovirus [38], [39], a HEV-C virus, has not been well described.

Transmission intensity of HEV usually varies with season with high transmission in summer, low transmission in winter, and intermediate transmission in spring and autumn. Chains of HEV71 transmission in Shandong province, represented by LY/SD lineage 1 and 2, were observed to have continued through the winter of 2007–2008. The subsequent 2008 isolates (FY/AH lineage) and 2009 isolates (SQ/HeN lineage 1 and 2) appear to be descendents of LY/SD lineage that further evolved via a common pathway identified by the *VP1* substitutions T3323C, T3104C, A2732G, C2693T, and C2660T. So chains of virus transmission of HEV71 continued to evolve and circulate during the low-transmission winter seasons in the Central Plain China.

Based on the similarity plot and bootscan analysis, C4a and C4b HEV71 strains are all recombinants, although there is no close match to a potential parent at the nucleotide level for the recombinant noncapsid sequences, it has the highest similarity to those of HEV-A other than HEV-B, C, and D, indicating that the recombinant noncapsid sequences could be classified into a HEV-A phylogeny. These findings demonstrate that during natural multiplication of HEV71 strains, they likely “trap” sequences from other HEV-A viruses, thereby producing new individual viruses that differ from the parental strains. However, the exact recombination counterpart of HEV-A could not be found because there is not sufficient data regarding the *P2* and *P3* sequences of the HEV-A in China or any other part of the world, but it may be assumed that genetic exchanges had occurred when the HEV71 strain co-circulated with other HEV-A during that time period in China. And, because all C4a and C4b are recombinants, so the relationship between recombination and neurovirulence are uncertain.

HEV71 continues to adapt to its hosts via nucleotide mutations, but such mutations often lead to the changes in the pathogenicity, patterns of prevalence, and clinical manifestations of HEV71 infection, complicating clinical diagnosis, treatment, vaccine development, and prevention and control. Therefore, dealing with HEV71 mutation and evolution is a great challenge for HFMD prevention and control efforts. Although the possible role of recombination in the phenotypic drift is not very clear, and it seems that the recombination events occurred before the differentiation of C4a and C4b evolutionary branches of HEV71, we propose that the recombination with HEV-A may not be essential and is not correlated with the phenotypic drift to higher neurovirulence and higher transmissibility, but it may be an indicator of the duration of viral circulation in the human community. Our research team is currently using reverse genetics methods to identify the key nucleotide and amino acid differences between evolutionary branches C4a and C4b (especially an A to C reversion at nt2503 in the *VP1* coding region, which caused amino acid substitution of VP1–10: Gln to His) in order to discover the important determinants of neurovirulence and the possible reasons for the repeated outbreaks of HFMD in China in recent years.

In conclusion, this study demonstrates that the high rate of HEV71 genomic evolution permits the use of comparative nucleotide sequencing to resolve the fine structure of HEV71 transmission within and between outbreaks. The evolutionary relationships among HEV71 infections determined from the combined sequence and epidemiological data reveal the underlying seasonal dynamics of HEV71 circulation. At least 5 HEV71 lineages were circulating in the Central Plain of China from 2007 to 2009, and the Shandong and Anhui lineages were found to have passed through the genetic bottleneck of the winter low-transmission season.

## Materials and Methods

### Viruses

This study did not involve human participants or human experimentation; the only human materials used were stool samples, throat swab samples, and vesicles collected from HFMD patients at the instigation of the Ministry of Health P. R. of China for public health purposes, and written informed consent for the use of their clinical samples was obtained from all patients involved in this study. This study was approved by the second session of the Ethics Review Committee of the Chinese Center for Disease Control and Prevention. The HEV71 strains used in this study were isolated between 2007 and 2009 from stool, throat swabs, or vesicles from HFMD patients from different geographical locations in the Shandong, Anhui, and Henan provinces of China (Table S1). Viruses were isolated from original clinical specimens by propagation in RD (human rhabdomyosarcoma) cells by conventional methods and then sequenced. To investigate the molecular epidemiology of HEV71 in Mainland China, 17 additional Chinese HEV71 sequences (obtained from the Guangdong, Chongqing, Shanghai, Zhejiang, Shanxi, Yunnan, and Hebei provinces between 1999 and 2010) and 19 international HEV71 sequences (obtained from the GenBank database) were also analyzed (Table S1).

### Determination of the complete *VP1* nucleotide sequences

Viral RNA was extracted from the viral isolates using a QIAamp Viral RNA Mini Kit (Qiagen, Valencia, CA, USA) and stored at −80°C until further use. The complete *VP1*/capsid region of each HEV71 strain was amplified by reverse transcriptase-polymerase chain reaction (RT-PCR) using in-house primers flanking the *VP1* region [16]. RT-PCR was performed with an Access RT-PCR Kit (Promega, USA) according to the instructions provided. The PCR products were purified using the QIAquick Gel Extraction Kit (Qiagen, Valencia, CA, USA), and the amplicons were bi-directionally sequenced using an ABI PRISM 3100 Genetic Analyzer (Applied Biosystems, Hitachi, Japan).

### Full-length genome sequencing of HEV71

The full-length genomes of 12 HEV71 strains from the HFMD patients in Shandong and Henan provinces were amplified and sequenced. The viral RNA was converted to cDNA by a random-priming strategy. The cDNA was amplified using the primers designed by multiple alignments of HEV71 genomes available in GenBank database (listed in Table 2). PCR products obtained were purified using the QIAquick Gel extraction kit (Qiagen). The amplicons were then bi-directionally sequenced using an ABI PRISM 3100 genetic analyzer (Applied Biosystems). 5′-segment sequences were determined by using a 5′-rapid amplification of cDNA ends (RACE) core set (Takara Biomedicals), according to the manufacturer's instructions.

**Table 2:** PCR and sequencing primers.

Primer	Nucleotide position (nt)	Primer sequence (5-3)	Orientation	Reference
0001S48^a^		GGGGACAAGTTTGTACAAAAAAGCAGGCTTTAAAACAGCTCTGGGGTT	Forward	[47]
HEV71-659A	640–659	CAATTGCTCTGTTGCACACC	Reverse	This study
EV/PCR-2	449–473	TCCGGCCCCTGAATGCGGCTAATCC	Forward	[48]
HEV71-1018A	999–1018	GTGATGGTGGAGTTGCCAAT	Reverse	This study
HEV71-863S	863–882	GGGCAAACAGAGTCTCAAGC	Forward	This study
HEV71-1948A	1929–1948	GCGCAGTCTCTCCATTAAGC	Reverse	This study
HEV71-1767S	1767–1786	GTTTCGGCACCTATTCTACC	Forward	This study
HEV71-2578A	2559–2578	TGGAGTGCTGGAACCTTACC	Reverse	This study
HEV71-VP1-S	2372–2391	GCAGCCCAAAAGAACTTCAC	Forward	[16]
HEV71-VP1-A	3435–3454	AAGTCGCGAGAGCTGTCTTC	Reverse	[16]
HEV71-3342S	3342–3351	CAGTCTGGGGCCATTTATGT	Forward	This study
HEV71-4345A	4326–4345	TGGAACTTGCGACAGAAGTG	Reverse	This study
HEV71-4181S	4181–4200	AGTACCAGCAGCCAAGGAGA	Forward	This study
HEV71-5207A	5188–5207	GGTGGGAGTTTCAGGAATGA	Reverse	This study
HEV71-5068S	5068–5087	CACAATCGAGGCTCTTTTCC	Forward	This study
HEV71-6195A	6175–6195	GCTGCCTCTTTGATGTACTCG	Reverse	This study
HEV71-5897S	5897–5916	CGCAGGCCTTAAAAGGAGTT	Forward	This study
HEV71-6749A	6730–6749	TGTGTGGTTGATTCCCTCAA	Reverse	This study
HEV71-6602S	6602–6621	TTTGCTCCCTGGTTCACTCT	Forward	This study
7500A^a^		GGGGACCACTTTGTACAAGAAAGCTGGG(T)_24_	Reverse	[47]

### Recombination analysis

Two nucleotide alignments were generated using the MEGA program (version 4.0; Sudhir Kumar, Arizona State University, Tempe, Arizona, USA) [40], [41]. The first alignment contains the genome sequences of a C4a evolutionary branch HEV71 strain (518-03F/SD/CHN/2007) and HEV-A prototype strains (CVA-2, 3, 4, 5, 6, 7, 8, 10, 12, 14, 16, HEV71, 76, 89, 90, 91 and 92); The second contains the genome sequences of a C4b evolutionary branch HEV71 strain (ShZh98/GD/CHN/1998) and HEV-A prototype strains. Once aligned, similarity plot and bootscan analysis were performed using Simplot program (version 3.5.1; Stuart Ray, Johns Hopkins University, Baltimore, Maryland, USA) [42].

### Phylogenetic and Bioinformatics analysis

Alignment of the complete *VP1* nucleotide sequences of the HEV71 isolates was performed using BioEdit Sequence Alignment Editor software (version 7.0.9; Tom Hall, North Carolina State University, Raleigh, North Carolina, USA) [43]. The sequences of *VP1* region were aligned and use an interactive and hierarchical multiple-logo visualization tool, Phylo-mlogo [44], base on nucleotide acids composition for grouping, and then the gene dataset for analysis were selected from each group. Phylogenetic trees were constructed by the neighbor-joining method using the MEGA program (version 4.0; Sudhir Kumar, Arizona State University, Tempe, Arizona, USA) [40], [41]. The branch lengths of the dendrogram were determined from the topologies of the trees and were obtained by majority rule consensus among 1000 bootstrap replicates. Bootstrap values greater than 80% were considered statistically significant for grouping.

### Reconstruction of the pathways of *VP1* sequence evolution of outbreak isolates

An evolutionary tree representing the pathways of HEV71 *VP1* evolution (which in turn represent transmission pathways) was constructed from the combined sequence and epidemiology data. The branches of the tree were constructed manually under the assumption that the observed substitutions were generated by the smallest possible number of mutational steps, and the tree was rooted to the earliest isolates. The topology of the manually constructed tree was confirmed by using phylogeny programs based upon neighbor-joining (MEGA) and maximum likelihood (PHYLO_WIN) algorithms [40], [41], [45].

### Evolutionary analysis based on the Bayesian Markov chain Monte Carlo method

The evolutionary rate and molecular clock phylogeny of the C4a and C4b branches of HEV71 were inferred using the Bayesian MCMC method in the BEAST 1.6.1 program [46], and the t_MRCA_ with 95% HPD was estimated. In order to reduce the computation load, sequences with high homogeneity and identical isolation years were deleted. Data were analyzed under both the HKY and the GTR nucleotide substitution models with gamma distribution of among-site rate variation. Two different models of rate variation among branches were implemented in our analysis: the strict clock and the UCLD relaxed molecular clock. Both constant and EG population size coalescents were used as tree priors. For each model, the MCMC chain was run for 30,000,000 steps and sampled every 1,000 steps.

### Nucleotide sequence accession numbers

The complete HEV71 *VP1* nucleotide sequences (891 nucleotides) that were determined in this study have been deposited in the GenBank database under the accession numbers JN256059 to JN256068, and JN835271 to JN835284. The full-length genomic sequences that were determined in this study have been deposited in the GenBank database under the accession numbers EU753365, EU753375, EU753384, EU753397, EU753398, EU753407, JN256059 to JN256064. The HEV71 isolates for which *VP1* nucleotide sequences or full-length genomic sequences were obtained or used in this study are described in the Table S1.

## Supporting Information

Table S1 **Genotypes, sources, and accession numbers, the year and the place of isolation, and associated pathological conditions of the HEV71 strains used to generate the HEV71 phylogenetic dendrograms.** Abbreviations of Chinese provinces: SD, Shandong; AH, Anhui; HeN, Henan; YN, Yunnan; SX, Shanxi; HeB, Hebei; ZJ, Zhejiang; CQ, Chongqing, SH, Shanghai; GD, Guangdong.
(DOC)
