# Supplementary material for: Emergence and Transmission Pathways of Rapidly Evolving Evolutionary Branch C4a Strains of Human Enterovirus 71 in the Central Plain of China
Source: PLoS One. 2011 Nov 18;6(11):e27895. doi: 10.1371/journal.pone.0027895 (PMC3220707; doi:10.1371/journal.pone.0027895)
Supplement: Table S1 — Genotypes, sources, and accession numbers, the year and the place of isolation, and associated pathological conditions of the HEV71 strains used to generate the HEV71 phylogenetic dendrograms. Abbreviations of Chinese provinces: SD, Shandong; AH, Anhui; HeN, Henan; YN, Yunnan; SX, Shanxi; HeB, Hebei; ZJ, Zhejiang; CQ, Chongqing, SH, Shanghai; GD, Guangdong. (DOC) [file pone.0027895.s001.doc]

**Table S1.** Supporting Information Table. Genotypes, sources, and accession numbers, the year and the place of isolation, and associated pathological conditions of the HEV71 strains used to generate the HEV71 phylogenetic dendrograms.

Abbreviations of Chinese provinces: SD, Shandong; AH, Anhui; HeN, Henan; YN, Yunnan; SX, Shanxi; HeB, Hebei; ZJ, Zhejiang; CQ, Chongqing, SH, Shanghai; GD, Guangdong.

| Strain | Genotype | Source | GenBank No. | The year of isolation | The place of isolation | Associated pathological conditions |
| --- | --- | --- | --- | --- | --- | --- |
| CVA16/G-10/RSA/1951 | // | GenBank | U05876 | 1951 | RSA | // |
| HEVi71/BrCr/USA/1970 | A | GenBank | U22521 | 1970 | USA | Severe |
| SB9508-SAR/MAS/2003 | C1 | GenBank | AY258301 | 2003 | MAS | // |
| 0915/USA/1987 | C1 | GenBank | AF009549 | 1987 | USA | // |
| 8M-6/AUS/1999 | C2 | GenBank | AF376109 | 1999 | AUS | // |
| 2641/AUS/1995 | C2 | GenBank | AF135947 | 1995 | AUS | // |
| 03/KOR/2000 | C3 | GenBank | AY125968 | 2000 | KOR | // |
| 97-56/HLJ/CHN/1997 | C3 | GenBank | AB115494 | 1997 | HLJ,CHN | // |
| 933V/VNM/2005 | C5 | GenBank | AM490161 | 2005 | VNM | // |
| 962T/VNM/2005 | C5 | GenBank | AM490162 | 2005 | VNM | // |
| AFP98111341/TAI/1998 | C4b | GenBank | DQ841953 | 1998 | TAI | // |
| 3254/TAI/1998 | C4b | GenBank | AF286531 | 1998 | TAI | // |
| N3340/TAI/2002 | C4b | GenBank | EU131776 | 2002 | TAI | // |
| 12242/THA/2008 | C4b | GenBank | FJ151494 | 2008 | THA | // |
| 17461/THA/2008 | C4b | GenBank | FJ151502 | 2008 | THA | // |
| ShZh98/GD/CHN/1998 | C4b | GenBank | AF302996 | 1998 | GD,CHN | Mild |
| F1/SH/CHN/2000 | C4b | GenBank | AB115490 | 2000 | SH,CHN | Mild |
| F2/SH/CHN/2000 | C4b | GenBank | AB115491 | 2000 | SH,CHN | Mild |
| H25/SH/CHN/2000 | C4b | GenBank | AB115492 | 2000 | SH,CHN | Mild |
| H26/SH/CHN/2000 | C4b | GenBank | AB115493 | 2000 | SH,CHN | Mild |
| ShZh01-3/GD/CHN/2001 | C4b | GenBank | AY895132 | 2001 | GD,CHN | Mild |
| SHH02-17/GD/SH/CHN/2002 | C4b | GenBank | AY547500 | 2002 | GD,CHN | Mild |
| ShZh02-62/GD/CHN/2002 | C4b | GenBank | AY895136 | 2002 | GD,CHN | Mild |
| ShZh03-106/GD/CHN/2003 | C4b | GenBank | AY895138 | 2003 | GD,CHN | Mild |
| ShZh04-3/GD/CHN/2004 | C4b | GenBank | AY895142 | 2004 | GD,CHN | Mild |
| E2004104/TAI/2004 | C4a | GenBank | DQ841964 | 2004 | TAI | // |
| E20051733/TAI/2005 | C4a | GenBank | DQ841971 | 2005 | TAI | // |
| 540V/VNM/2005 | C4a | GenBank | AM490151 | 2005 | VNM | // |
| SI01/THA/2006 | C4a | GenBank | EF203407 | 2006 | THA | // |
| 1827-Yamagata/JPN/2007 | C4a | GenBank | AB433890 | 2007 | JPN | // |
| 1897-Yamagata/JPN/2007 | C4a | GenBank | AB433891 | 2007 | JPN | // |
| CQ03-1/CQ/CHN/2003 | C4a | GenBank | AY547501 | 2003 | CQ,CHN | Mild |
| ZJ03-1/ZJ/CHN/2003 | C4a | GenBank | AY905614 | 2003 | ZJ,CHN | Mild |
| 518-01F/SD/CHN/2007 | C4a | GenBank | EU753363 | 2007 | SD,CHN | Mild |
| 518-03F/SD/CHN/2007 | C4a | GenBank | EU753365 | 2007 | SD,CHN | Severe |
| 519-02F/SD/CHN/2007 | C4a | GenBank | EU753366 | 2007 | SD,CHN | Mild |
| 521-04T/SD/CHN/2007 | C4a | GenBank | EU753369 | 2007 | SD,CHN | Severe |
| 521-09F/SD/CHN/2007 | C4a | GenBank | EU753372 | 2007 | SD,CHN | Mild |
| 521-18S/SD/CHN/2007 | C4a | GenBank | EU753375 | 2007 | SD,CHN | Severe |
| 521-25F/SD/CHN/2007 | C4a | GenBank | EU753379 | 2007 | SD,CHN | Mild |
| 522-04T/SD/CHN/2007 | C4a | GenBank | EU753384 | 2007 | SD,CHN | Mild |
| 522-08T/SD/CHN/2007 | C4a | GenBank | EU753386 | 2007 | SD,CHN | Mild |
| 522-17T/SD/CHN/2007 | C4a | GenBank | EU753389 | 2007 | SD,CHN | Mild |
| 523-05T/SD/CHN/2007 | C4a | GenBank | EU753397 | 2007 | SD,CHN | Severe |
| 523-07T/SD/CHN/2007 | C4a | GenBank | EU753398 | 2007 | SD,CHN | Mild |
| 523-11T/SD/CHN/2007 | C4a | GenBank | EU753402 | 2007 | SD,CHN | Mild |
| TC03F/SD/CHN/2007 | C4a | GenBank | EU753407 | 2007 | SD,CHN | Severe |
| TC08F/SD/CHN/2007 | C4a | GenBank | EU753409 | 2007 | SD,CHN | Severe |
| TC16T/SD/CHN/2007 | C4a | GenBank | EU753413 | 2007 | SD,CHN | Mild |
| TC23F/SD/CHN/2007 | C4a | GenBank | EU753417 | 2007 | SD,CHN | Mild |
| TC24F/SD/CHN/2007 | C4a | GenBank | EU753418 | 2007 | SD,CHN | Mild |
| FY17.08-1/AN/CHN/2008 | C4a | GenBank | EU703812 | 2008 | AH,CHN | Fatal |
| FY17.08-2/AN/CHN/2008 | C4a | GenBank | EU703813 | 2008 | AH,CHN | Fatal |
| FY17.08-3/AN/CHN/2008 | C4a | GenBank | EU703814 | 2008 | AH,CHN | Fatal |
| FY17.08-5/AN/CHN/2008 | C4a | GenBank | GQ121418 | 2008 | AH,CHN | Mild |
| FY17.08-6/AN/CHN/2008 | C4a | GenBank | GQ121419 | 2008 | AH,CHN | Severe |
| FY17.08-7/AN/CHN/2008 | C4a | GenBank | GQ121420 | 2008 | AH,CHN | Severe |
| FY17.08-8/AN/CHN/2008 | C4a | GenBank | GQ121421 | 2008 | AH,CHN | Severe |
| FY17.08-10/AN/CHN/2008 | C4a | GenBank | GQ121417 | 2008 | AH,CHN | Mild |
| FY18.08-1/AN/CHN/2008 | C4a | GenBank | GQ121427 | 2008 | AH,CHN | Fatal |
| FY19.08-1/AN/CHN/2008 | C4a | GenBank | GQ121433 | 2008 | AH,CHN | Mild |
| FY19.08-6/AN/CHN/2008 | C4a | GenBank | GQ121441 | 2008 | AH,CHN | Mild |
| FY19.08-7/AN/CHN/2008 | C4a | GenBank | GQ121424 | 2008 | AH,CHN | Severe |
| G224-882F/HeN/CHN/2009 | C4a | This study | JN835271 | 2009 | HeN,CHN | Severe |
| G227-885F/HeN/CHN/2009 | C4a | This study | JN835272 | 2009 | HeN,CHN | Severe |
| G252-910F/HeN/CHN/2009 | C4a | This study | JN835273 | 2009 | HeN,CHN | Severe |
| G256-914F/HeN/CHN/2009 | C4a | This study | JN835274 | 2009 | HeN,CHN | Severe |
| G283-922F/HeN/CHN/2009 | C4a | This study | JN835275 | 2009 | HeN,CHN | Severe |
| G288-927F/HeN/CHN/2009 | C4a | This study | JN256059 | 2009 | HeN,CHN | Fatal |
| G306-945F/HeN/CHN/2009 | C4a | This study | JN835276 | 2009 | HeN,CHN | Severe |
| G333-972F/HeN/CHN/2009 | C4a | This study | JN256060 | 2009 | HeN,CHN | Severe |
| G348-987F/HeN/CHN/2009 | C4a | This study | JN835277 | 2009 | HeN,CHN | Severe |
| G392-1031F/HeN/CHN/2009 | C4a | This study | JN835278 | 2009 | HeN,CHN | Severe |
| G398-1037F/HeN/CHN/2009 | C4a | This study | JN256061 | 2009 | HeN,CHN | Severe |
| G400-1039F/HeN/CHN/2009 | C4a | This study | JN835279 | 2009 | HeN,CHN | Severe |
| G405-1045F/HeN/CHN/2009 | C4a | This study | JN835280 | 2009 | HeN,CHN | Severe |
| G443-1083F/HeN/CHN/2009 | C4a | This study | JN835280 | 2009 | HeN,CHN | Severe |
| G523-1157T/HeN/CHN/2009 | C4a | This study | JN835282 | 2009 | HeN,CHN | Severe |
| G541-1175F/HeN/CHN/2009 | C4a | This study | JN835283 | 2009 | HeN,CHN | Severe |
| M183-1176F/HeN/CHN/2009 | C4a | This study | JN256062 | 2009 | HeN,CHN | Fatal |
| M184-1177F/HeN/CHN/2009 | C4a | This study | JN835284 | 2009 | HeN,CHN | Severe |
| M186-1179F/HeN/CHN/2009 | C4a | This study | JN256063 | 2009 | HeN,CHN | Severe |
| M188-1181F/HeN/CHN/2009 | C4a | This study | JN256064 | 2009 | HeN,CHN | Severe |
| HeB-132/HeB/CHN/2010 | C4a | This study | JN256065 | 2010 | HeB,CHN | Mild |
| HeB-310/HeB/CHN/2010 | C4a | This study | JN256066 | 2010 | HeB,CHN | Severe |
| SX006/SX/CHN/2010 | C4a | This study | JN256067 | 2010 | SX,CHN | Mild |
| T126/YN/CHN/2010 | C4a | This study | JN256068 | 2010 | YN,CHN | Fatal |
